# African Organization for Research and Training in Cancer: position and vision for cancer research on the African Continent

**DOI:** 10.1186/s13027-016-0110-9

**Published:** 2016-12-06

**Authors:** J. Olufemi Ogunbiyi, D. Cristina Stefan, Timothy R. Rebbeck

**Affiliations:** 1 AORTIC Research Committee, University of Ibadan, Ibadan, Nigeria; 2 AORTIC Research Committee, Walter Sisulu, Umtata, South Africa; 3 International Prevention Research Institute, Lyon, France; 4 AORTIC Research Committee, Dana Farber Cancer Institute and Harvard TH Chan School of Public Health, Boston, USA; 5 Department of Pathology, College of Medicine, University of Ibadan, Ibadan, Nigeria

**Keywords:** African Cancer Burden, Infections and cancer in Africa, Diagnosis of cancer, Access to healthcare, Cancer research

## Abstract

The African Organization for Research and training in Cancer (AORTIC) bases the following position statements on a critical appraisal of the state on cancer research and cancer care in Africa including information on the availability of data on cancer burden, screening and prevention for cancer in Africa, cancer care personnel, treatment modalities, and access to cancer care.

## Background

### Cancer is a major public health concern In Africa

Cancer is a leading cause of death worldwide. About half of the annual incident cancer cases occur in the developing world. There were an estimated 14.1 million new cancer cases and 8.2 million cancer related deaths in 2012 [4]. Of these, there were 715,000 incident cancer cases and 542,000 deaths in Africa, with increasing incidence of breast and prostate cancers. The incidence of cancer is therefore increasing worldwide and the continuing global demographic and epidemiologic transitions signal an ever-increasing cancer burden over the next decades, particularly in low- and middle-income countries (LMIC). Africa is expected to carry a major cancer burden by year 2030 [4]. Incidence rates of 1.27 million with 0.97 million deaths are estimated in 2030 for Africa.

Cancer in Africa has many unique features. As shown in Table 1, the leading cancers in Africa include many of those that are common around the world, but also include cancers that are less common in high-income countries and reflect patterns of cancer more commonly seen in low- and middle-income countries (LMIC). In addition, the distribution of cancer types varies substantially within Africa, and these differ compared to the cancer type distribution in other parts of the world, with a high proportion of infection related cancers in many areas in Africa [8]. In men, prostate cancer is the leading cancer in most parts of Africa, similar to that in many other parts of the world. However, liver cancer is the leading cancer in large sections of West Africa, Kaposi Sarcoma is the leading cancer in Southeast Africa, and esophageal cancer is the leading cancer in Botswana. In addition, while breast cancer is the leading cancer in women in many parts of Africa, cervical cancers predominate in West Africa and parts of East and Central Africa [4]. Kaposi’s sarcoma was the second largest contributor to the cancer burden in sub-Saharan Africa. The AFs for infection varied by country and development status—from less than 5% in the USA, Canada, Australia, New Zealand, and some countries in western and northern Europe to more than 50% in some countries in sub-Saharan Africa [8].

**Table 1:** Leading cancers in men and women in Africa (Source: Globocan 2012)

Site	Deaths	Adjusted Rate^a^	Cumulative Risk^b^
Men			
Prostate	42,802	17.0	1.5
Liver	37,012	11.8	1.3
Lung	19,430	7.0	0.8
Kaposi sarcoma	16,343	4.9	0.5
Colorectum	15,102	5.1	0.6
Non-Hodgkin lymphoma	15,021	4.3	0.4
Oesophagus	14,702	5.3	0.6
Stomach	12,000	4.1	0.5
Leukaemia	11,638	3.1	0.3
Bladder	9362	3.5	0.4
Pancreas	6424	2.3	0.3
Lip, oral cavity	6083	2.1	0.2
Brain, nervous system	5415	1.6	0.2
Larynx	4258	1.5	0.2
Kidney	4155	1.1	0.1
Nasopharynx	3478	1.1	0.1
Hodgkin lymphoma	2834	0.8	0.1
Other pharynx	2631	0.9	0.1
Multiple myeloma	2573	0.9	0.1
Melanoma of skin	1543	0.5	0.1
Women			
Breast	63,160	17.3	1.8
Cervix uteri	60,098	17.5	2
Liver	19,045	5.6	0.6
Colorectum	14,270	4.2	0.5
Ovary	13,085	3.8	0.4
Non-Hodgkin lymphoma	11,427	2.9	0.3
Oesophagus	10,542	3.3	0.4
Stomach	9801	3.0	0.3
Leukaemia	9483	2.4	0.3
Kaposi sarcoma	9184	2.2	0.2
Lung	7653	2.4	0.3
Pancreas	5280	1.6	0.2
Brain, nervous system	4581	1.2	0.1
Lip, oral cavity	4258	1.3	0.1
Corpus uteri	4030	1.3	0.2
Kidney	4014	0.9	0.1
Thyroid	3979	1.3	0.2
Bladder	3906	1.2	0.1
Gallbladder	2796	0.9	0.1
Multiple myeloma	2494	0.8	0.1

^a^Age-adjusted rate per 100,000 population

^b^Cumulative Risk ages 0–74 in %

#### Cancer in Africa

**Fig. 1:**
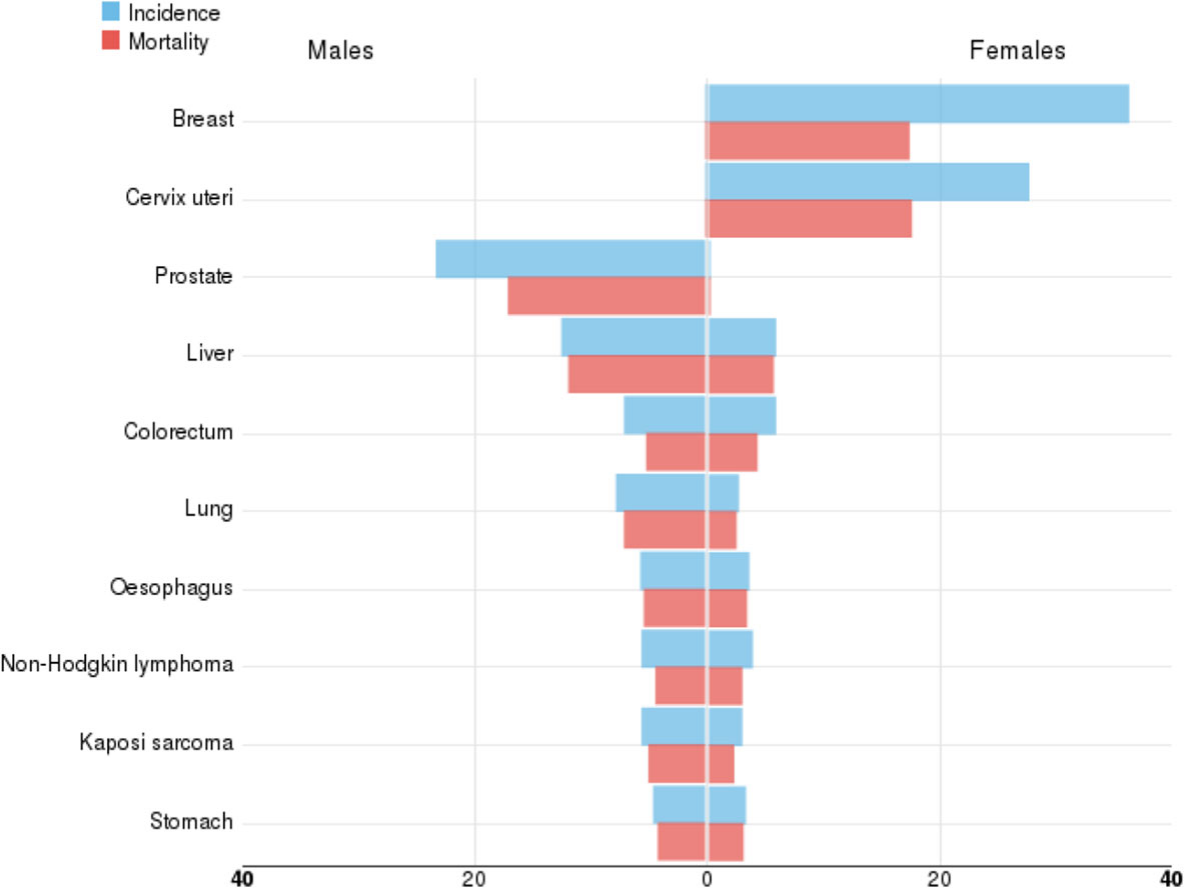
Age-standardized incidence and mortality rates for the leading cancers in Africa (Source GLOBOCAN 2012)

Unlike other parts of the world, for many cancers, incidence and mortality rates in Africa are very similar (Fig. 1). Five-year survival trends have shown wide differences across the continents with variably significant improvements in some cancers in different developed countries but much less so in developing countries [3]. The high case-fatality rate in Africa can be attributed to a wide range of factors, including the following:

##### Delayed diagnosis

Lack of early and accurate diagnosis is a challenge to appropriate care. More than 80% of patients in Africa are diagnosed at advanced stages of cancer. Radiology facilities are too few to diagnose the population in need [6]. Inadequate pathology leads to wrong diagnosis and patients may receive inappropriate treatment [1, 2]. Scarcity of care providers and researchers is a problem in pathology training, and many countries have fewer than one pathologist for every million people [2]. Opportunities for prevention are widely under-utilized. For example, cervical cancer is the leading cause of cancer death for women in 40 of 48 countries in sub-Saharan Africa, yet many countries have limited screening services (PAP test) and HPV vaccination.

##### Access to healthcare

Problems and challenges for cancer control in Africa exist at every step of the patient’s journey. Awareness for cancer is lower in comparison with other infectious disease priorities. Cancer is often seen as a disease caused by spiritual curses, and as such cancer cases are often referred to healers or shamans for traditional or spiritual treatment. Health care providers in rural areas lack training on cancer, often misdiagnosing cancer as other illness. Lack of data on cancer prevalence and trends in Africa and historical focus on communicable diseases decrease government efforts on cancer research and treatment.

##### Availability of treatment modalities

High quality treatment is difficult due to limited healthcare sources and low affordability. In 2015, the estimated shortage of health care professionals (792,000) will cost $2.2+ billion annually in the 31 African countries [10, 11]. The current number of physicians practicing in Africa (145,000) represents 5% of the European total (2,877,000). Treatment access is also limited: ~22% of the 54 African countries have no access to anti-cancer therapies. Barriers to treatment include significant out-of-pocket expenses. Out-of-pocket health expenditure is estimated to push many people globally into dire poverty when treatment costs are substantially higher than income. Finally, there is a constant threat to the clinician pool due to ‘brain drain’. More than half of 168 medical schools surveyed reported losing between 6 to 18% of teaching staff to emigration in the last 5 years [7]. It will be critical to attract African health care personnel to more attractive settings with better salaries, working conditions, career paths and support.

##### End of life care

End of life care is a particularly critical domain for cancer control in Africa given the late presentation of many cancers and limited treatment opportunities. Cancer is diagnosed at such a late stage that treatment is no longer effective, leaving palliative care as the only option for reducing suffering. Inaccurate forecasting for highly-controlled medications has historically lead to shortages of critical pain relief options. Home-based care options are limited for African patients, especially outside capital cities. Rural families may view cancer as curse and therefore do not want to treat patient. Not all rural health facilities are authorized to stock powerful pain medications necessary to properly reduce human suffering associated with late stage cancer

### Why is cancer research important in Africa?

As described above, the unique pattern of cancers, cancer etiologies, and limitations in early detection, diagnosis and treatment in Africa suggest that priorities for cancer control (including prevention and treatment) are likely to be different than in other parts of the world. Therefore, strategies for cancer control in Africa require that data to be generated are relevant to African populations. Currently, cancer research in Africa is limited in a number of important ways. First, the number and quality of cancer registries and of trained cancer registry staff is inadequate to provide information on the cancer burden in Africa. Without this basic information, planning for clinical and public health needs in Africa is not optimal. Secondly, there are few cancer advocates and trained community health workers to inform the public and policy-makers about cancer. Thirdly, the number of oncologists and other cancer clinical specialists remains inadequate to deal with the current cancer burden. The situation doesn’t appear to improve as the projected number of oncologists and other cancer specialists continues to fall short in meeting the requirements to control the growing cancer burden in Africa. There are also relatively few cancer researchers to generate the knowledge base that may be required to create cancer control strategies in Africa. These include cancer epidemiologists, statisticians, scientists, public health experts, health economists, behavioral scientists, and others.

AORTIC has decided to address these problems by acknowledging that the cancer burden in Africa will continue to rise, data on cancer in Africa are extremely limited, there is poor funding for cancer research, there is inadequate research infrastructure in most of Africa, and cancer researchers are few, mostly senior people who are also otherwise engaged in “clinical medicine or governance.

### The AORTIC vision for cancer research in Africa

AORTIC has developed a cancer plan for Africa that began with the Dakar Declaration in 2011 (http://www.aortic-africa.org/index.php/news/aortic-dakar-declaration-press-release/) and has evolved with the publication of the Cancer Plan for the African Continent 2013–2017 (http://www.aortic-africa.org/images/uploads/AORTIC_CANCER_PLAN.pdf). These documents form the basis for the implementation of cancer control and research efforts by AORTIC in Africa. AORTIC continues to implement, evaluate, and extend these statements through its ongoing activities. The AORTIC Research Committee was formed to lead the planning and implementation of the cancer research initiatives outlined in these two documents. The guiding principles of the Research Committee were that cancer research in Africa should be impactful (i.e., able to inform science and clinical practice globally, while informing African public health and policy needs locally) and sustainable (i.e., led by Africans involved in global collaborative teams). This plan is premised on the fact that cancer research provides the evidence base on which prevention, control, and treatment strategies must be built. Components of this plan include improved policy and funding support, improved knowledge of cancer is Africa, awareness of cancer burden, clinical oncology infrastructure and improved cancer health systems, and cancer prevention and control.

The implementation of this plan includes the development of regional infrastructure that will foster cancer research; increase the quality and quantity of research workforce focused on cancer control in Africa; and promote and support translational research throughout the cancer control continuum across crosscutting issues, including communications, surveillance, social determinants of health, genetic testing, decision-making, dissemination of evidence-based interventions, quality of cancer care, epidemiology and measurement.

AORTIC has developed tangible products and activities to assist in the implementation of the general concepts of this plan. First, AORTIC produced a *Handbook for Cancer Research in Africa* that provides a general outline of Africa-specific research approaches for cancer [9]. Topics that were identified as being key to the success of cancer research in Africa that were included in this volume were basic research principles, career considerations, developing and maintaining research partnerships, responsible conduct of research, research funding, community engagement research, biosampling and biobanking, pathology, data management and analysis, clinical trials, research advocacy, and research dissemination.

Second, as specified in the cancer plan, AORTIC has initiated research training workshops on cancer in Africa. The first of these will be held in January 2017 in Cape Town, South Africa in collaboration with the American Association for Cancer Research. The target audience will be both established researchers who wish to develop cancer-specific projects as well as new investigators and trainees. The topics identified for the *Handbook* were carried over as the main workshop content areas.

Third, an African Cancer Research Alliance (ACRA) has been initiated. The goal of the ACRA is to develop the expertise, resources, and infrastructure that can lead to impactful investigator-initiated cancer research in Africa. The ACRA will share and develop research methods and technology that are optimally suited for research in Africa; create partnerships among institutions and investigators who can undertake collaborative research; facilitate research projects that will impact our understanding of cancer worldwide and impact clinical service and public health in Africa. In addition to these immediate goals, ACRA will improve research infrastructure in Africa, establish a trained African cancer research workforce, and generate data that impact on the health of populations in Africa and worldwide. The implementation of ACRA will involve identification of research “hubs” that have general capacity for cancer research including epidemiology, laboratory methods, clinical capacity, and other features. Characteristics of these hubs are being developed and hubs will be identified throughout Africa.

There are a number of examples of research networks upon which the ACRA network will be built, and from which research hubs could be identified. Three existing prostate cancer networks serve as a focus around which urological cancer research may be formed: the Men of African Descent and Carcinoma of the Prostate (MADCaP) network, The African Caribbean Cancer Consortium (AC3), and the Prostate Cancer Transatlantic Consortium (CaPTC). Similarly, the African Consortium on Cervical Cancer Control Research (COFAC-Col) and the African Breast Cancer Consortium can serve as foci around which ACRA networks and hubs will be established.

**Fig. 2:**
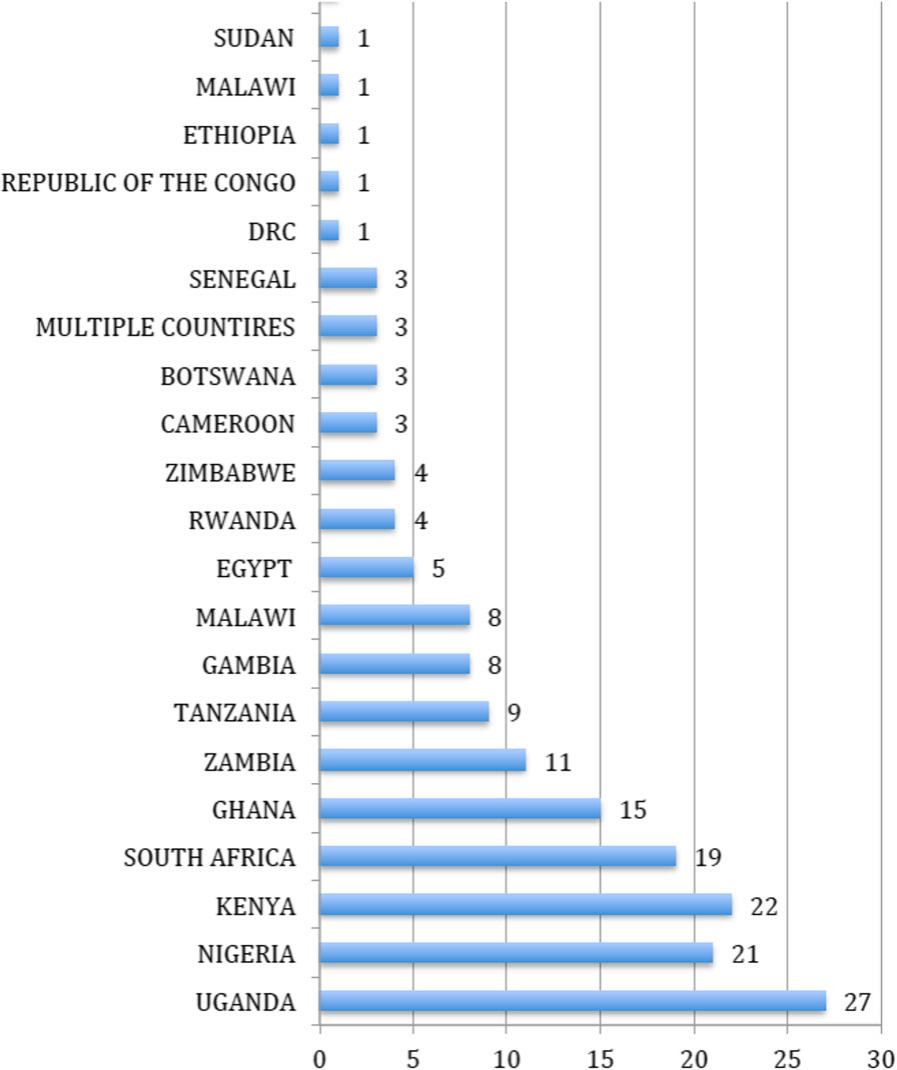
Countries with Cancer Research Projects as identified in the AORTIC Cancer Research Registry (http://mendel2.med.upenn.edu:8080/AfricaProject/MapView.jsp)

Finally, AORTIC has developed a registry of researchers and research projects through the African Cancer Research Registry (http://mendel2.med.upenn.edu:8080/AfricaProject/MapView.jsp). Figure 2 provides a summary of the countries in which cancer projects have been identified and included in registry This registry provides the potential for African cancer researchers to identify related projects and colleagues with similar interests. The data in this registry are also included in the Global Cancer Project Map (http://globalonc.org/Projects/global-cancer-project-map/) developed by Global Oncology. Ongoing research initiatives are being identified and will be included as found. However, in the future all data will be incorporated directly into the Global Cancer Project Map, which will serve as the central repository for this information in the future.

While ACRA will serve as the AORTIC focus for the development of cancer research activities in Africa, it will also strive to work with other existing partners. These include funders such as the US National Cancer Institute, the International Union Against Cancer (UICC), the American Association for Cancer Research (AACR), and the American Society for Clinical Oncology (ASCO). In addition, ACRA will work with the AORTIC Education and Training Committee to foster training opportunities as research capacity is built.

### Areas of focus

Data shown in Table 1 identify the leading cancer sites in Africa. These include prostate, liver, lung cancers and Kaposi sarcoma in men, and breast, cervical, liver, colorectal and ovarian cancers in women. In addition, childhood cancers, many of which are treatable or even curable, are of interest because of the high rates of mortality that remains for African children diagnosed with these tumors. The ACRA will focus much of its efforts in the development of research projects and infrastructure that address these most common cancers to maximally impact on the burden of cancer in Africans. While the data on cancer incidence and mortality rates are not as well captured in Africa as in the US, it is clear from recent data that many of the most common cancers in high income countries are also the most common in Africa, including prostate cancer, breast cancer and cervical cancer [4, 5]. These research activities can include determining optimal screening and treatment choices for prostate, breast, and cervical cancers; understanding the relative contributions of genetic, lifestyle, and environmental factors in the development and progression of breast and prostate cancer in Africa; determining the influence of emigration on breast and prostate cancer mortality comparing Africans in the diaspora to indigenous Africans; and investigating the HPV types and other complimentary risk factors that are peculiar to Africa. Other work will address unique needs in Africa, including research on vaccine production against HBV and HCV; development of new non-invasive tests for biomarkers of hepatocellular carcinoma using serological antibodies, proteomics and genomics technology; investigate lung cancer patterns and other causal risk factors apart from smoking in Africa; and explore the role of genetics as an independent risk factor for lung cancer in the African population. Thus, the mission of ACRA will be to promote inter- and intraregional collaborative research that will lead to improved cancer control in Africa.

### Going forward

**Fig. 3:**
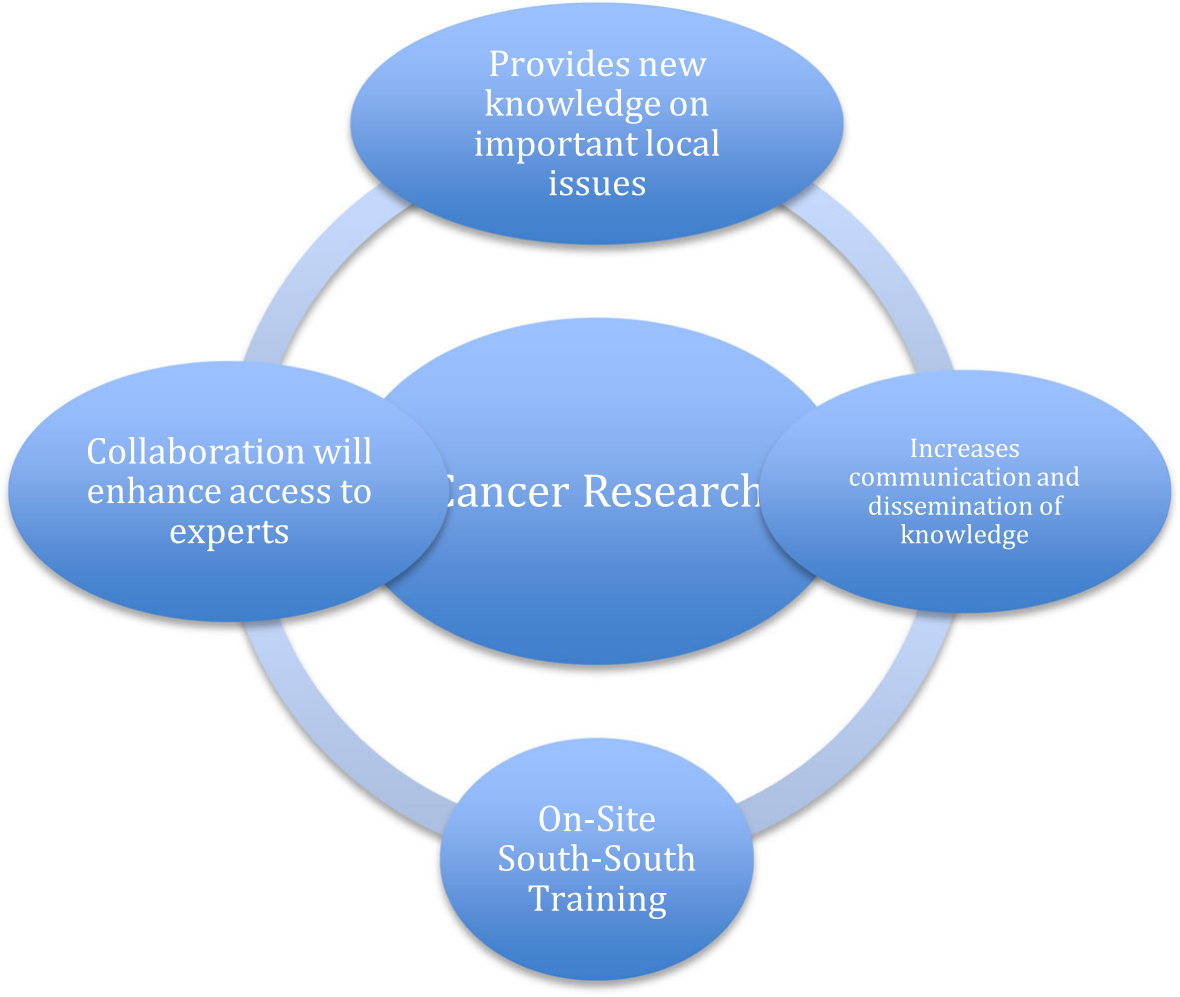
Anticipated benefits of Cancer Research Development in Africa

The AORTIC vision for cancer research in Africa is to provide knowledge that will inform cancer prevention and control in Africa that will reduce the number of deaths from cancer and improve the quality of life of cancer patients, survivors and caregivers. Research serves a critical need to generate knowledge around which clinical, public health, and policy can be developed for cancer in Africa. As shown in Fig. 3, AORTIC and its ACRA initiative will provide knowledge on important local issues, increase communication and dissemination of this knowledge, provide training in situ for research capacity building in Africa, and will enhance access to experts needed to develop this capacity. The implementation of this plan includes development of regional infrastructure that will foster cancer research; increase the quality and quantity of research workforce focused on cancer control in Africa; and promote and support translational research throughout the cancer control continuum across crosscutting issues, including communications, surveillance, social determinants of health, genetic testing, decision-making, dissemination of evidence-based interventions, quality of cancer care, epidemiology and measurement.
